# Knowledge, Attitudes, and Practices Toward Contraceptive Methods Among Female Undergraduate Students of Chiang Mai University, Thailand: A Cross-Sectional Survey

**DOI:** 10.1089/whr.2024.0126

**Published:** 2025-03-05

**Authors:** Pattaraporn Charussangsuriya, Jutarat Siri, Tanawat Jantra, Panisa Suebsai-on, Theera Tongsong, Sasivimol Srisukho

**Affiliations:** Department of Obstetrics and Gynecology, Faculty of Medicine, Chiang Mai University, Chiang Mai, Thailand.

**Keywords:** contraception methods, knowledge, attitudes, practices, undergraduate students

## Abstract

**Background:**

Unintended pregnancies are associated with unsafe abortions and maternal deaths. Undergraduates are at risk of unexpected pregnancy due to changes in their lives. Adequate knowledge and attitudes toward contraceptive uses are essential to help prevent pregnancy.

**Objective:**

To assess sex activity, knowledge, attitudes, and practices of contraception methods among female undergraduates at Chiang Mai University, Thailand.

**Methods:**

Self-administered anonymous questionnaires were answered by female undergraduate students via an online platform. The questionnaire consisted of four parts (sociodemographic and contraception use, knowledge of contraceptive methods, attitudes, and sources of information.).

**Results:**

A total of 475 women responded to the questionnaire. Of them, 29.2% had sex experience, with significantly lower prevalence among the students in health sciences faculties, when compared with those of nonhealth sciences (20.6% vs. 40.2%; *p*-value <0.001). The mean ± standard deviation scores of knowledge of contraceptive methods was 12.84 ± 4.59 (range, 0–24), indicating a medium level. There were only 15.1% of participants who were categorized as a high level, while many participants (86.9%) had a positive attitude toward contraceptive methods. Most participants gained contraceptive knowledge from online content from the internet and social media.

**Conclusion:**

Nearly 30% of the female university students had sex experience, lower than that in most previous studies, with significantly lower prevalence in the group of health sciences faculties. Most female university students had a positive attitude toward contraception uses; however, their knowledge about contraception was relatively limited. Finally, most contraceptive methods used were relatively less effective and theoretically at risk for unintended pregnancy.

## Introduction 

In 2022, the World Health Organization (WHO) announced a World Family Planning strategy to increase the number of women who use effective methods of contraception.^1^ Moreover, contraception has demonstrated additional benefits in preventing sexually transmitted infections (STIs) and unintended pregnancy.^2^ A global comparative analysis in 2015–2019 estimates that incidence of unintended pregnancy and abortion in Thailand was nearly 40%.^3^ Undergraduates are at risk of unintended pregnancy due to changes in their lives during their educational journey.^3^ Focusing on Thai culture, the undergraduate period should be considered as the most changing period in their lives. Before going to college, students usually live with their parents. After enrolling as freshmen at university, most students must leave home and live in university dormitories or private accommodations. The prevalence of unintended pregnancy among university students may be higher than expected. For instance, the rate of unintended pregnancies among unmarried, sexually active college students in mainland China is as high as 17.7%.^4^ As a result, unintended pregnancies among university students, who are in late adolescence or their early 20s, are expected to be relatively high and present a significant challenge, not only disrupting their educational paths but also complicating the balance of parenthood.

Moreover, adolescent pregnancy is associated with higher risks of adverse pregnancy outcomes, especially in low- and middle-income countries.^5^ Specifically, pregnancy during the academic year has a great impact not only on individual health but also on social well-being. Therefore, adequate knowledge and attitudes toward contraceptive methods are essential to help prevent pregnancy.^6^ Additionally, a Cochrane Database systematic review reported that a combination of educational and contraceptive interventions among adolescents appears to reduce unintended pregnancy.^7^

The cross-sectional study in China revealed that female college students who became unintentionally pregnant were 14.9%, and inadequate knowledge of using condoms was a factor associated with unintended pregnancies among sexually active undergraduates.^4^

A survey from Bhutan found that contraceptive use among university students was low and they had average knowledge and a good attitude toward STI.^8^ Also, a systematic review reported that adolescents from low- and middle-income countries face a lack of access to useful information about contraception and abortion.^9^

Since sexual behavior among female undergraduate students varies significantly across the world, influenced by factors such as culture, religion, lifestyle, and socioeconomics, it is essential for each population to develop its own data to understand the magnitude of the issues and devise appropriate strategies for improving women’s health during their educational years. Accordingly, we conducted this study to assess the prevalence of sexual activity and the knowledge, attitudes, and practices regarding contraceptive methods among female undergraduates at Chiang Mai University, Thailand. We expect that the results of this study will uncover the influences and barriers to contraceptive use and help in developing reproductive health strategies to prevent unintended pregnancies during the university period.

## Methods

The study was ethically approved by the institutional review boards, Faculty of Medicine, Chiang Mai University (Research ID: OBG-2566-0059). Informed consent was obtained from all participants. This study mainly focused on knowledge of contraception methods, attitudes toward contraception, and sources of information.

### Participants and eligibility criteria

This study is a descriptive cross-sectional study, conducted on female undergraduate students aged 18–26years, who were studying for a bachelor’s degree at Chiang Mai University, located in Northern Thailand between July 1, 2023, and August 31, 2023. The female undergraduate students were voluntarily selected, using a simple random sampling method. The participants were randomly selected at the end of the classes and during the break time. Self-administered anonymous questionnaires were answered by participants via an online platform. Participants, who did not understand Thai language, were excluded.

### Study questionnaires

The questionnaire consisted of four parts. Part I included questions of sociodemographic data (age, level of education, faculty, income, occupation, current marital status, sexual activity, history of contraception use, and current contraception method). Part II comprised 24 questions about the knowledge of contraceptive methods including natural methods, oral contraceptive pills, condoms, contraceptive injections, intrauterine devices, and contraceptive implants. The correct answer was scored as 1, whereas the “wrong and unsure answer” was scored as 0. Out of 24 questions on contraceptive methods, women whose scores were >17 were arbitrarily classified as having “good,” 12–17 were classified as having “medium,” and less than 11 were considered as having “poor.” Part III dealt with 15 questions designed to investigate participants’ attitudes toward contraceptive use. The answers of attitude about contraceptives were classified according to a 5-level Likert rating scale. The total score was 75 points. Participants had a positive attitude when they got a score of 56 points or more, whereas those with a score of less than 56 points were considered a negative contraceptive attitude. The final part was to survey the sources of information. Prior to the study, the questionnaire of part II and part III in Thai version were contemplated, discussed, and revised until consensus among four researchers. The content validity was confirmed by three gynaecologists, Department of Obstetrics and Gynecology, Chiang Mai University. The experts rated each of the 39 questions as either “relevant” or “not relevant.” The Item-level Content Validity Index (I-CVI) was calculated for each item, and the CVI was calculated by taking the average proportion of 24 questions about knowledge concerning contraception methods and 15 questions about attitudes. The CVI was 1 for both parts of the questionnaire. The reliability was tested in 10 women with the same characteristics as the participants. The Cronbach’s alpha coefficient for the reliability was 0.82.

The main outcomes were (1) the prevalence of sexual activity, (2) scores of knowledges of contraceptive methods, (3) percentage of positive attitude to contraception, (4) sources of contraceptive knowledge, (5) percentages of sex experience among female undergraduates, and (6) percentages of each contractive method used.

### Statistical analysis

All data were collected in an electronic database and subsequently analyzed, using the statistical package for the social sciences (SPSS) software version 26.0 (IBM Corp. Released 2019. IBM SPCSS Statistics for Windows, Armonk, NY: IBM Corp). Descriptive statistics were used to analyze demographic data, which were expressed as numbers with percentages, mean with standard deviation, or median with interquartile range, as suitable. In comparisons of various parameters between the participants in the health science group and those in the nonhealth sciences group, Student’s *t* test, chi-square test, and Fisher’s exact test were used as appropriate. Pearson correlation and Spearman’s rho correlation coefficient were used to test the correlation between attitudes and knowledge score of contraception, as suitable. A *p* value of <0.05 was defined as statistical significance.

## Results

### Sociodemographic characteristics of the participants

Four hundred and 75 women responded to the questionnaire, and demographic data are presented in Table 1. The age of the participants was 19.41 ± 1.30 years (mean ± standard deviation [SD]). The proportion of participants who studied in faculties of health sciences was 56.0% (266/475). Approximately 70% of the participants were freshmen and sophomores. Two-thirds of the participants had monthly income of less than 10,000 Thai baths. The majority of undergraduates, 431 (90.7%), were Buddhist. About 13.2% were still living with their parents, while 86.8% lived independently in dormitory. Of all, 139 (29.2%) of the participants had experience of sexual activity. Notably, the prevalence of students with experience of sexual activity was significantly lower in the group of health sciences than the other (20.6% vs. 40.2%, *p*-value <0.001). Concerning the contraceptive methods, condom was the most common method of contraception (92.8%), followed by emergency contraceptive pill (41.0%). The frequencies of each method used were not significantly different between the two groups, as presented in Table 1. Notably, among those who experienced sexual intercourse, only 7.9% used long-acting reversible contraceptive (LARC) methods (intrauterine devices, injections, and implants), and the rates were comparable between both groups, as presented in Table 1. Interestingly, the number of undergraduates with the habits of alcohol drinking, smoking, and having part-time jobs was significantly lower in the group of health sciences faculties than those in the non-health sciences faculties.

**Table 1.** Demographic Data (*n* = 475)

Characteristics	Total (*n* = 475)	Health science (*n* = 266)	Others (*n* = 209)	*p*-Value
	Mean ± SD			
Age (years)	19.41 ± 1.30	19.54 ± 1.39	19.25 ± 1.24	0.018^a^
BMI (kg/m^2^)	20.62 ± 3.49	20.76 ± 3.36	20.44 ± 3.64	0.313^a^
	*n* (%)			
Education level				0.101^b^
Year 1	214 (45.1%)	105 (52.2%)	109 (50.9%)	
Year 2	127 (26.7%)	78 (29.3%)	49 (23.4%)	
Year 3	78 (16.4%)	48 (18.0%)	30 (14.4%)	
Year 4	46 (9.7%)	27 (10.2%)	19 (9.1%)	
Year 5	6 (1.3%)	5 (1.9%)	1 (0.5%)	
Year 6	4 (0.8%)	3 (1.1%)	1 (0.5%)	
Income per month				0.083^b^
<10,000 bath	366 (77.0%)	196 (63.7%)	170 (81.3%)	
10,001–20,000 bath	77 (20.5%)	64 (24.1%)	33 (15.8%)	
>20,001 bath	12 (2.5%)	6 (2.3%)	6 (2.9%)	
Religion				0.150^b^
Buddhism	431 (90.7%)	244 (91.7%)	187 (89.5%)	
Christ	22 (4.6%)	14 (5.3%)	8 (3.8%)	
Muslim	2 (0.4%)	0 (0.0%)	2 (1.0%)	
None	20 (5.0%)	8 (3.0%)	12 (5.7%)	
Accommodation				**0.019** ^b^
Living with parents	63 (13.3%)	28 (10.5%)	35 (16.7%)	
Private dormitory/condominium	244 (51.4%)	131 (49.2%)	113 (54.1%)	
University dormitory	168 (35.4%)	107 (40.2%)	61 (29.2%)	
Alcohol drinking	259 (54.5%)	126 (47.4%)	133 (63.6%)	**<0.001** ^b^
Smoking	17 (3.6%)	3 (1.1%)	14 (3.6%)	**0.002** ^c^
Part-time job	39 (12.0%)	14 (5.3%)	25 (12.0%)	**0.008** ^b^
Status				**0.012** ^b^
Single	350 (73.7%)	208 (78.2%)	142 (67.9%)	
In relationship	125 (29.3%)	58 (21.8%)	67 (32.0%)	
Sexual intercourse	139 (29.2%)	55 (20.6%)	84 (40.2%)	**<0.001** ^b^
Contraception methods				
Fertility awareness-based method	32 (23.0%)	11 (20.0%)	21 (25.0%)	0.494
Coitus interruptus	43 (30.9%)	16 (29.1%)	27 (32.1%)	0.703
Condom	129 (92.8%)	51 (92.7%)	78 (92.9%)	0.977
Emergency pills	57 (41.0%)	21 (38.2%)	36 (42.9%)	0.584
Oral combined pills	39 (28.1%)	14 (25.5%)	25 (29.8%)	0.580
Hormonal injections	1 (0.7%)	0 (0%)	1 (1.2%)	1.000
Intrauterine devices	1 (0.7%)	0 (0%)	1 (1.2%)	1.000
Hormonal implants	9 (6.5%)	3 (5.5%)	6 (7.1%)	1.000
Surgical fertilization	1 (0.7%)	1 (1.8%)	0 (0%)	0.396

Bold represent statistical significance of *p*-values.

HS: Health sciences included the Faculty of Medicine, Faculty of Dentistry, Faculty of Pharmacy, Faculty of Nursing, Faculty of Associated Medical Science, and Faculty of Veterinary Medicine.

Other: Non-health sciences included the Faculty of Agriculture, Faculty of Law, Faculty of Business administration, Faculty of Humanities, Faculty of Political Science and Faculty of Public Administration, Faculty of Fine Arts, Faculty of Science, Faculty of Engineering, Faculty of Economics, Faculty of Architecture, Faculty of Social Sciences, Faculty of Mass Communication, Faculty of Agroindustry, Faculty of Education, and Faculty of College of Art, Media and Technology.

^a^ Student’s *t* test.

^b^ Chi-square test.

^c^ Fisher’s exact test.

BMI, body mass index; SD, standard deviation.

### Knowledge and attitudes of contraceptive methods

The results of the knowledge and attitudes of contraceptive methods are shown in Table 2. The mean ± SD knowledge score of contraceptive methods among female undergraduate students was 12.84 ± 4.59 (range, 0–24), indicating a medium level. Only 15.1% of participants were categorized as a high knowledge group, whereas 47.4% and 37.5% were categorized as the moderate and low knowledge group, respectively. Regarding subcategories of contraceptive methods, the mean ± SD of knowledge scores about natural and condoms were 2.61 ± 2.10 and 3.06 ± 2.22, respectively, higher than the knowledge scores of the others. The majority of participants (86.9%) had a positive attitude toward contraceptive methods. While the overall average score was moderate (12.84), among participants who had a good total knowledge score (18–24), 98.61% also had positive attitude scores. Furthermore, there was a weakly positive correlation between attitude and knowledge (*r* = 0.253, *p*-value <0.001). Despite this, the finding showed no significant relationship between age, body mass index, income, religion, status, and knowledge/attitude score regarding contraceptive methods (Table 3).

**Table 2.** Knowledge and Attitudes Score of Contraceptive Methods (*n* = 475)

Variable	Score range	Mean ± SD	*n* (%)
Total knowledge score	0–24	12.84 ± 4.59	
Good	18–24	19.58 ± 1.53	72 (15.1%)
Medium	12–17	14.41 ± 1.66	225 (47.4%)
Poor	0–11	8.13 ± 2.83	178 (37.5%)
Total knowledge score based on the methods^a^			
Natural contraception	0–5	2.61 ± 2.10	
Oral contraceptive pills	0–5	0.94 ± 1.55	
Condom	0–5	3.06 ± 2.22	
Contraceptive injection	0–3	0.56 ± 0.93	
Intrauterine devices	0–3	0.69 ± 1.10	
Contraceptive implant	0–3	0 .94 ± 1.55	
Total attitude score	42–74	63.40 ± 5.98	
Positive	57–74	65.16 ± 3.98	413 (86.9%)
Negative	42–56	51.72 ± 3.32	62 (13.1%)

^a^ Note that, the questionnaire consisted of 24 questions, with a total possible score of 24 points, distributed as follows: 5, 5, 5, 3, 3, and 3 points for each of the six methods. Each participant’s possible score ranged from 0 to 24, with one point awarded for each correct answer.

**Table 3.** Correlation Between Demographic Characteristics, Attitude, and Knowledge regarding Contraceptive Methods (*n* = 475)

Variable	Knowledge score		Attitude score	
	Correlation	*p*-Value	Correlation	*p*-Value
Age	0.078^b^	0.089	0.008	0.864
BMI	0.024^b^	0.603	0.064	0.166
Education level	0.056^a^	0.223	0.030	0.519
Income	0.077^a^	0.095	−0.51	0.270
Religion	0.012^a^	0.797	−0.085	0.064
Accommodation	0.002^a^	0.969	0.053	0.248
Alcohol drinking	0.039^a^	0.398	0.012	0.797
Smoking	0.097^a^	**0.034**	0.102	**0.026**
Part-time job	0.023^a^	0.615	0.001	0.983
Status	0.044^a^	0.344	−0.087	0.058
Sexual intercourse	0.008^a^	0.860	0.034	0.457
Contraception using	−0.793^a^	0.396	0.193	**0.023**
Attitude Score	0.253^a^	**<0.001**	1	1

Bold represent statistical significance of *p*-values.

^a^ Pearson correlation.

^b^ Spearman’s rho correlation coefficient.

To compare knowledge and attitudes scores among health science and nonhealth science students, there were no significant differences across all levels between health science and non-health science members (Table 4).

**Table 4.** Knowledge and Attitudes and Sources of Information When Comparing Between Health Sciences Group and Others (*n* = 475)

Variable	Mean ± SD			*p*-Value^a^
	Total (*n* = 475)	HS (*n* = 266)	Others (*n* = 209)	
Total knowledge score	12.84 ± 4.59	13.11 ± 4.46	12.50 ± 4.73	0.517
Good	19.58 ± 1.53	19.65 ± 1.703	19.48 ± 1.27	0.714
Medium	14.41 ± 1.66	14.45 ± 1.64	14.35 ± 1.68	0.356
Poor	8.13 ± 2.83	8.47 ± 2.62	7.73 ± 3.04	0.277
Total attitude score	63.40 ± 5.98	63.76 ± 5.62	62.94 ± 6.38	0.294
Positive	65.16 ± 3.98	65.16 ± 4.20	65.14 ± 3.92	0.393
Negative	51.72 ± 3.32	52.34 ± 3.14	51.18 ± 3.43	0.351

^a^ Independent *t* tests.

### Sources of information about contraception method

**FIG. 1.**
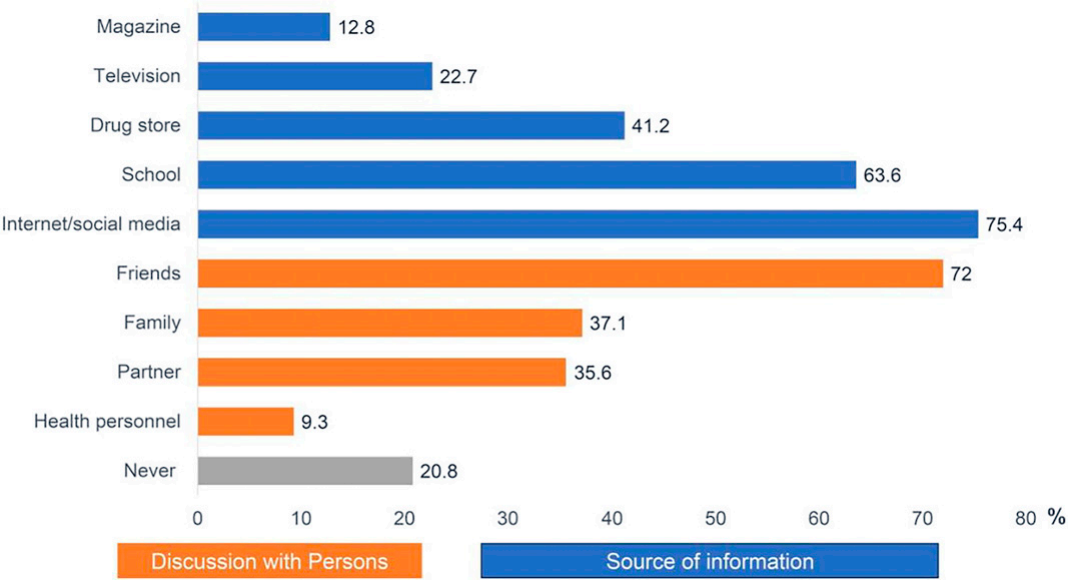
Sources of information about contraception method (*n* = 475).

Regarding sources of information about contraception methods, most responders (79.2%; *n* = 376) indicated that they sought contraceptive data from a wide variety of sources. Most participants gained contraceptive knowledge from online content from the internet and social media. The top three social media that they mentioned were Twitter, Facebook, and Google. Additionally, they had learned from high school and asked for data from drug stores, 63.6% and 41.2%, respectively. A total of 342 (72.0%) reported that they also discussed contraception methods with friends. Only 37.1% and 35.6% got information from their family and their partners, respectively. Interestingly, one-fifth never found any information on contraception methods. Other sources were also cited by the participants, as shown in Figure 1.

## Discussion

This study focused on undergraduate students (the mean age of 19.41 years) who are in education age, including late adolescent and young adult age, because this group of women is in the period of transitional lifestyle, changing from school life to university life, from life under closely taken care of by the parents to be more independent life with more exposure to sexual relationship and vulnerable to have sexual intercourse. Importantly, most of sexual exposures in this group of women are in personal secret life instead of exposed relation as family life. Accordingly, the knowledge, attitude, and practice of contraception methods among this group of people are essential and should be uncovered for proper strategies to reduce problems associated with unplanned unwanted pregnancies. This study found that 29.2% of our female university students in 2023 already have had sexual activity. This is much less than that reported in most previous studies. For examples, 70.4% of female undergraduate students in the two Universities in Tanzania in 2013,^10^ nearly 70% of university students in Uganda in 2016,^11^ and nearly 77.6% of university students in Nigeria in 2009^12^ already had sexual intercourse. These data suggest that sexual behavior among female undergraduate students around the world can be markedly different, probably associated with different cultures, religions, lifestyles, socioeconomic status, and the like. Therefore, to take the right action for improvement of women health in their education age, each population should develop its own data to identify the magnitude of the problems, leading to development of the right strategy.

Moreover, this study found that only 15% of our undergraduates had good knowledge about contraception methods. Therefore, health educators and professionals should provide accurate information and education about contraception methods to students. More education on contraception among our university students is still in need to enhance the compliance of contraception use and reduce the proportion of unwanted pregnancies. Note that, our findings are different from results from other university settings. For example, 44.68% of study participants in Ethiopia had good knowledge of modern contraceptive methods.^13^ This high level of knowledge may be attributed to the mass campaign on contraception previously carried out throughout the country.

Not all unintended pregnancies are undesired. In fact, many women may accept the pregnancy, take good care of it, and successfully manage both their university lives and personal outcomes. Therefore, they should be approached with proper counseling. However, since unintended and unwanted pregnancies are of significant concern for female university students, the most effective contraceptive methods are preferred. Nevertheless, the intentional application of a patient-centered reproductive justice framework, combined with the use of a shared decision-making model, is the recommended approach for providing supportive contraceptive counseling and care to help patients achieve their reproductive goals.^14–18^ Although LARCs, such as intrauterine devices and subdermal progestin-releasing implants, should be considered the primary choice for sexually active university students, they should also be encouraged to use contraception that aligns with their individual preferences. Thus, interventions that promote translation of this knowledge into proper sexual and reproductive health practices are seriously needed in our population. Note that, regardless of the chosen effective contraceptive method, the use of condom is still necessary for prevention of STI.

Condoms were the most preferred method of contraception among our participants. Our findings were similar to those reported in studies elsewhere in both developed and developing countries.^11,12,19,20^ Besides preventing unplanned pregnancies, condoms, when used correctly and consistently, are among the most effective methods of preventing most STIs. Universities may develop a proper policy to enhance accessibility of condoms to support safe sex without compromising our good culture of having sex with responsibility at appropriate time of age.

A notable finding in the present study was the high rate of positive attitudes toward contraception methods among university participants (86.9%). This was close to 85% of the participants showed a positive attitude toward contraceptive use in one previous report from Saudi Arabia.^21^ Regarding attitudes, these findings are important because if health care providers encourage them and provide information about contraception methods, these are very important factors that may ultimately improve understanding and levels of knowledge. Moreover, these will influence undergraduate students’ compliance with contraceptive use. Certainly, poor attitude toward contraceptive use could be a barrier of understandings and accurate knowledge, probably leading to unprotected sex.

Several studies have investigated whether the main sources of information about contraception were from friends, radio, and school (39.5%−73.7%, 36%, and 24%), respectively.^10,12,22^ Unsurprisingly, with consistent results, the top three sources of information about contraception were social media, friends, and school. Collectively, our previous findings suggest a need for promoting knowledge of contraception use. We found that these sources were the preferred choices among undergraduates to increase their understanding of contraceptive techniques.

To our knowledge, this survey is the first study based on a knowledge, attitudes, and practices survey regarding contraceptive methods among female undergraduate students in Northern Thailand. This study was conducted using a validated questionnaire investigating knowledge, attitudes, and practices. Moreover, all questions were answered quite completely by the participants. Specific issues that had high percentages of misconception or inadequate knowledge should be useful for health care providers to explain, discuss, or clarify with the women. Thus, in order to motivate undergraduates to use contraception correctly, we recommend educating them and undergoing follow-ups during university periods.

Interestingly, university students of the health sciences had a significantly lower prevalence of sex experience than those of non-health sciences. The main reasons of such a difference were unclear. Nevertheless, this might be associated with the different nature of more learning activities, which needed more concentration on study. Also, the habits vulnerable to have more sexual exposures such as alcohol drinking, smoking, and having part-time jobs were significantly lower in the students of health sciences when compared with those in the non-health sciences faculties. Additionally, the number of students living in the university dormitory, which is likely less exposed to have sexual relationship, was significantly greater in the health sciences group. However, knowledge and attitudes toward contraceptive use and sources of information were comparable between the two groups.

Limitations of this study are as follows: (1) the study population included only students from one university. Therefore, the findings of this study might not be extrapolated to all female students of other universities. (2) It is possible that some of the answers concerning sexual activity might not be perfectly reliable because this is a very sensitive and personal issue of their secret life, though this survey was based on anonymous approach. (3) The information on regular or occasional sexual activity was not assessed. This may be important for contractive choices and probably occasional sexual activity was associated with condom use, which is the most common practice method among women with not regular sex.

## Conclusions

The main interesting findings are as follows: (1) While the prevalence of sexual activity among female university students markedly varies around the world, the prevalence in Chiang Mai University is approximately 30%. (2) Such a prevalence among the students in non-health science group is two times as much as that among those in health science group. Thus, even in the same university, the strategy to improve women health may need the different levels of strengthening. (3) Most female university students had a positive attitude toward contraception uses; however, their knowledge about contraception was relatively limited. (4) Most contraceptive methods used were relatively less effective and theoretically at risk for unintended pregnancy. LARC methods, which are preferably recommended for young women, are very rare in this study. New knowledge on this issue needs to be encouraged. Because of a lack of or inadequate knowledge of contraception methods, several undergraduates may not adhere to the use of contraception. Hence, the policy to improve knowledge of contraception methods is also crucial. Establishing user-friendly reproductive and sexual health services may help improve student engagement in contraception methods.

## Data Availability

The datasets used and/or analyzed during the current study are available from the corresponding author upon reasonable request.

## Abbreviations Used

CVI: Item-level Content Validity Index= I
LARC: Long-acting reversible contraceptive
SD: Standard Deviation
SPSS: Statistic Package for the Social Sciences
STIs: Sexually Transmitted Infections
WHO: World Health Organization
